# Mechanisms downstream of reverse transcription reduce serum levels of HBV DNA but not of HBsAg in chronic hepatitis B virus infection

**DOI:** 10.1186/s12985-015-0447-5

**Published:** 2015-12-09

**Authors:** Simon B. Larsson, Sebastian Malmström, Charles Hannoun, Gunnar Norkrans, Magnus Lindh

**Affiliations:** Department of Infectious Diseases, Institute of Biomedicine, Sahlgrenska Academy, University of Gothenburg, Gothenburg, Sweden

**Keywords:** HBV DNA, HBsAg, cccDNA, Replication, pgRNA

## Abstract

**Background:**

Hepatitis B virus (HBV) DNA in serum of chronically infected patients declines by 3–4 log_10_ units at loss of HBe antigen (HBeAg) from serum. The mechanisms behind this decline, and the much smaller decline of surface antigen (HBsAg) levels, are still not well known. The aim of this study was to get a better understanding of this process by analysing both serum and intrahepatic markers of HBV replication.

**Methods:**

Levels of HBV DNA and HBsAg in serum, and covalently closed circular DNA (cccDNA), pregenomic RNA (pgRNA) and S-RNA and total intrahepatic HBV DNA (ihDNA) in liver biopsies from 84 chronically infected patients (16 positive and 68 negative for HBeAg) were analysed.

**Results:**

Lower HBV DNA levels within HBeAg-positive stage reflected lower levels of cccDNA and pgRNA with strong correlation. In HBeAg-negative patients, ihDNA levels were greater and HBV DNA levels in serum lower than expected from pgRNA levels. A lower HBV DNA/HBsAg ratio corresponded with lower pgRNA/cccDNA (*p* < 0.01) and higher S-RNA/cccDNA (*p* < 0.0001) ratios, suggesting that in HBeAg-negative patients transcription of pgRNA, but not of S-RNA, becomes suppressed.

**Conclusions:**

The marked reduction of HBV DNA in serum after loss of HBeAg appears to be due to combined reduction of cccDNA, pgRNA and yet unidentified mechanisms downstream of reverse transcription. Such mechanisms include faster clearance of circulating virus or blocked secretion of virions, the latter supported by the observed relative increase of ihDNA in HBeAg-negative patients. The smaller reduction of S-RNA than of pgRNA partly explains why HBsAg remain high in the HBeAg-negative stage, supporting the possibility of HBsAg synthesis from integrated HBV DNA.

## Background

Despite the availability of an effective vaccine, chronic hepatitis B virus (HBV) infection remains an important cause of liver cirrhosis and hepatocellular carcinoma, in particular in East Asia and Sub-Saharan Africa [1]. In clinical diagnostics, HBV DNA levels in serum are the most important marker for the assessment of prognosis and treatment outcome during chronic HBV infection. In early stage of chronic infection HBV DNA usually persists at high levels, above 7–8 log_10_ IU/mL, for many years, often decades. Later in life the HBV DNA levels decline when the immune response evolves to more efficiently suppress viral replication. The greatest reduction of HBV DNA is observed at loss of hepatitis B e antigen (HBeAg) from the blood, a critical step that typically represents a transition from high to low replicative stage, usually also conferring clinical improvement [2]. Mechanisms that explain this pronounced reduction of viremia are not fully clarified. Part of the reduction of HBV DNA in serum is a result of reduced intrahepatic levels of covalently closed circular DNA (cccDNA), and of the pregenomic RNA (pgRNA), a transcript from cccDNA that is reverse transcribed to minus strand HBV DNA during formation of new viral particles [3]. The cccDNA minichromosome is also template for transcripts that are translated into hepatitis B surface antigens (HBsAg) embedded in the envelope of both virions and so-called subviral particles (SVP), of which the latter are produced in great excess [4].

In diagnostics, detection of HBsAg has been used as the basic test for identification of HBV infection for decades. Recently, quantification of HBsAg has come into focus as a complement to HBV DNA levels for monitoring both the natural course of infection and the response to treatment [5]. Levels of HBsAg, which mainly represent the levels of SVP [4], are more stable over time than HBV DNA, with a much smaller decline during the course of infection. This difference is in particular striking during loss of HBeAg when HBV DNA declines by 3–4 log_10_ or even more, whereas the HBsAg levels often are reduced by only 1 log_10_ [6–8]. As a consequence, the ratio between these two serum markers (HBV DNA/HBsAg) decreases when HBV DNA reaches very low or even undetectable levels while HBsAg remains at significant levels [9]. Previous studies indeed indicate that reduced transcription of pgRNA from cccDNA may contribute to the decline in HBV DNA [10]. It is however not well known to what extent such effects are specific for pgRNA, i.e. are not also reducing S-RNA. In the present study we explored the relations between HBV markers in serum and liver biopsies from both HBeAg-positive and HBeAg-negative patients, searching for explanations to the strong reduction of HBV DNA in serum and the shift in HBV DNA/HBsAg ratio that occur in parallel with loss of HBeAg. The contribution of different steps of replication to the reduced serum levels of HBV DNA and HBsAg is discussed.

## Results

### Patient characteristics

A summary of patient characteristics is found in Table 1. HBeAg-negative patients had significantly lower levels of viral markers than HBeAg-positive patients except for a higher ratio of S-RNA to cccDNA.

**Table 1:** Summary of patient characteristics and virological findings

	HBeAg+ *n* = 16	HBeAg– *n* = 68	*P* value
Age, years, median, (range)	27 (16–60)	34 (18–59)	0.02
Gender (M/F)	11/5	40/28	0.57
Genotype (A/B/C/D)	3/4/2/7	15/7/3/43	–
Serum HBV DNA^a^	8.30 ± 0.38	4.30 ± 0.14	<0.0001
Serum HBsAg^b^	4.40 ± 0.18	3.41 ± 0.11	<0.0001
HBV DNA/HBsAg	3.90 ± 0.29	0.89 ± 0.14	<0.0001
ALT/ULN, median, (range)	1.37 (0.68–8.6)	0.72 (0.24–12.38)	<0.001
cccDNA^c^	−0.70 ± 0.20	−2.67 ± 0.10	<0.0001
S RNA^c^/cccDNA^c^	1.65 ± 0.20	2.73 ± 0.11	<0.0001
pgRNA^c^/cccDNA^c^	2.04 ± 0.12	1.50 ± 0.11	<0.01
pgRNA^c^/S RNA^c^	0.39 ± 0.23	−1.22 ± 0.07	<0.0001

Virological values as mean ± SEM. *P* values by Mann-Whitney *U* test for age and ALT, *t* test for virological parameters and Fisher’s exact test for gender

^a^log_10_ cp/mL

^b^log_10_ IU/mL

^c^log_10_ copies/cEq (cell equivalent), ALT/Upper Limit of Normal

### Reduction of viral replication prior to loss of HBeAg

**Fig. 1:**
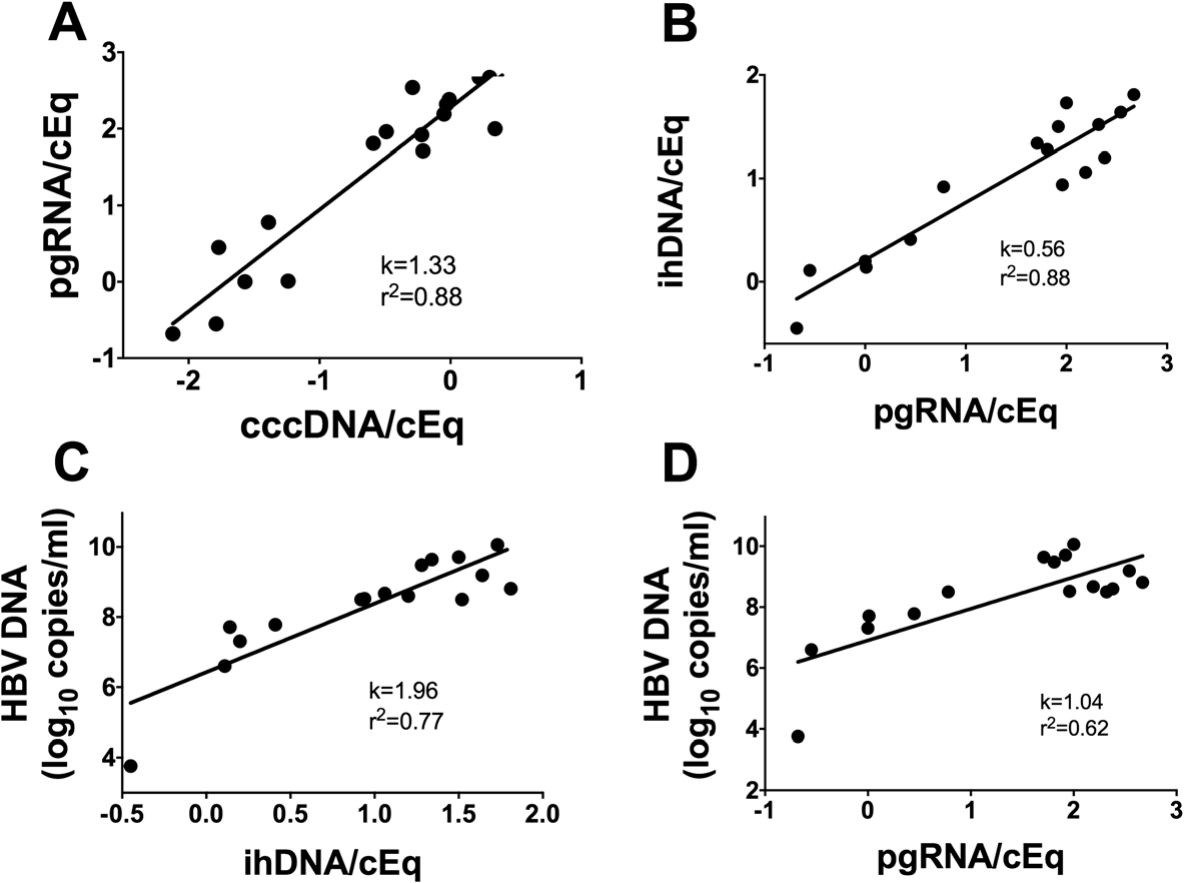
Correlations between intrahepatic HBV DNA (ihDNA) cccDNA, pregenomic RNA (pgRNA) and serum HBV DNA within HBeAg-positive patients. pgRNA vs. cccDNA (**a**), ihDNA vs. pgRNA (**b**), HBV DNA vs. ihDNA (**c**), and HBV DNA vs. pgRNA (**d**). All values are log_10_ copies/cell equivalent (cEq) if not stated otherwise

In order to study this, the levels of HBV markers within HBeAg-positive patients, and correlations between them, were analysed. As shown in Fig. 1a, the levels of cccDNA spanned over more than 2 log_10_ units within this group. Levels of pgRNA correlated well with cccDNA (R^2^ = 0.88), with a regression line slope of 1.33, indicating that a 2 log_10_ decline of cccDNA corresponded to a 2.7 log_10_ decline of pgRNA. The correlations in Fig. 1b (ihDNA and pgRNA) and C (serum HBV DNA and ihDNA) were also strong among HBeAg-positive patients, with R^2^ = 0.88 and R^2^ = 0.77. The slopes were however different, indicating that ihDNA declines less than pgRNA (k = 0.56), whereas HBV DNA in serum declines more than ihDNA (k = 1.96). As a result, the correlation between pgRNA and HBV DNA in serum had k = 1.04 (R^2^ = 0.62), indicating that until loss of HBeAg, reduction of HBV DNA in serum is achieved mainly by mechanisms that lead to reduced pgRNA levels (Fig. 1d).

### Viral markers after loss of HBeAg

**Fig. 2:**
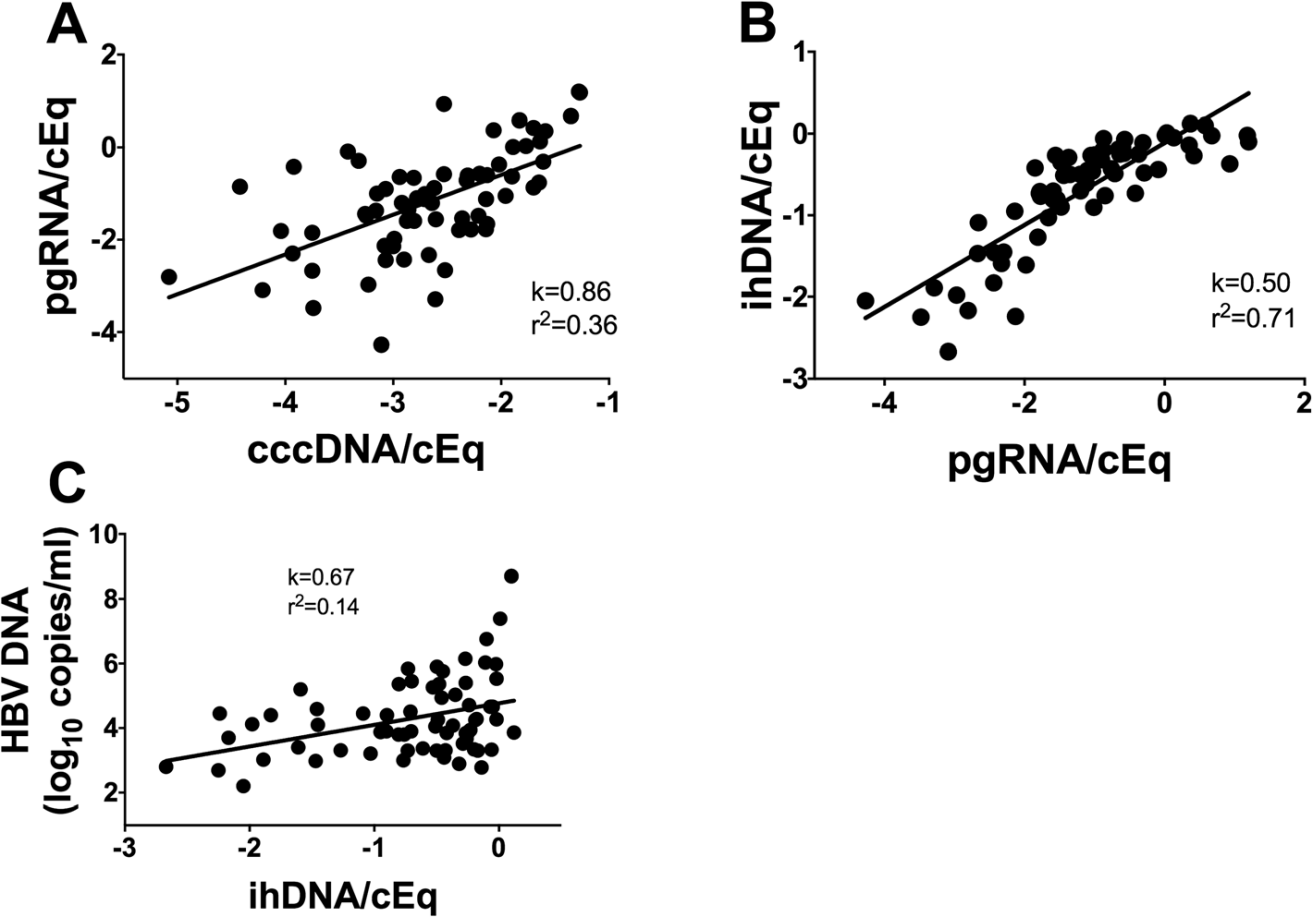
Correlations between intrahepatic HBV DNA (ihDNA), cccDNA, pregenomic RNA (pgRNA) and serum HBV DNA within HBeAg-negative patients. pgRNA vs. cccDNA (**a**), ihDNA vs pgRNA (**b**), and HBV DNA vs. ihDNA (**c**). All values are log_10_ copies/cell equivalent (cEq) if not stated otherwise

To some extent this can be studied by analysing correlations between viral markers within HBeAg-negative patients. Figure 2a shows that the correlation between cccDNA and pgRNA was poorer (R^2^ = 0.36) than in HBeAg-positive stage, indicating that the regulation of pgRNA levels becomes more variable after loss of HBeAg. By contrast, the correlation between pgRNA and ihDNA (Fig. 2b) remained strong and with a similar slope in HBeAg-negative (R^2^ = 0.72, k = 0.50) as in HBeAg-positive patients, suggesting that the rate of reverse transcription is unaltered after loss of HBeAg. The correlation between ihDNA and serum HBV DNA was on the other hand remarkably different in HBeAg-negative patients (Fig. 2c) with low R^2^ (0.14). In particular, the levels of HBV DNA in serum were much lower in a large proportion of patients than one would expect from the pgRNA and ihDNA level (if the correlations had been the same as in HBeAg-positive stage), suggesting that viremia is supressed by mechanisms acting after reverse transcription or after secretion into the blood.

**Fig. 3:**
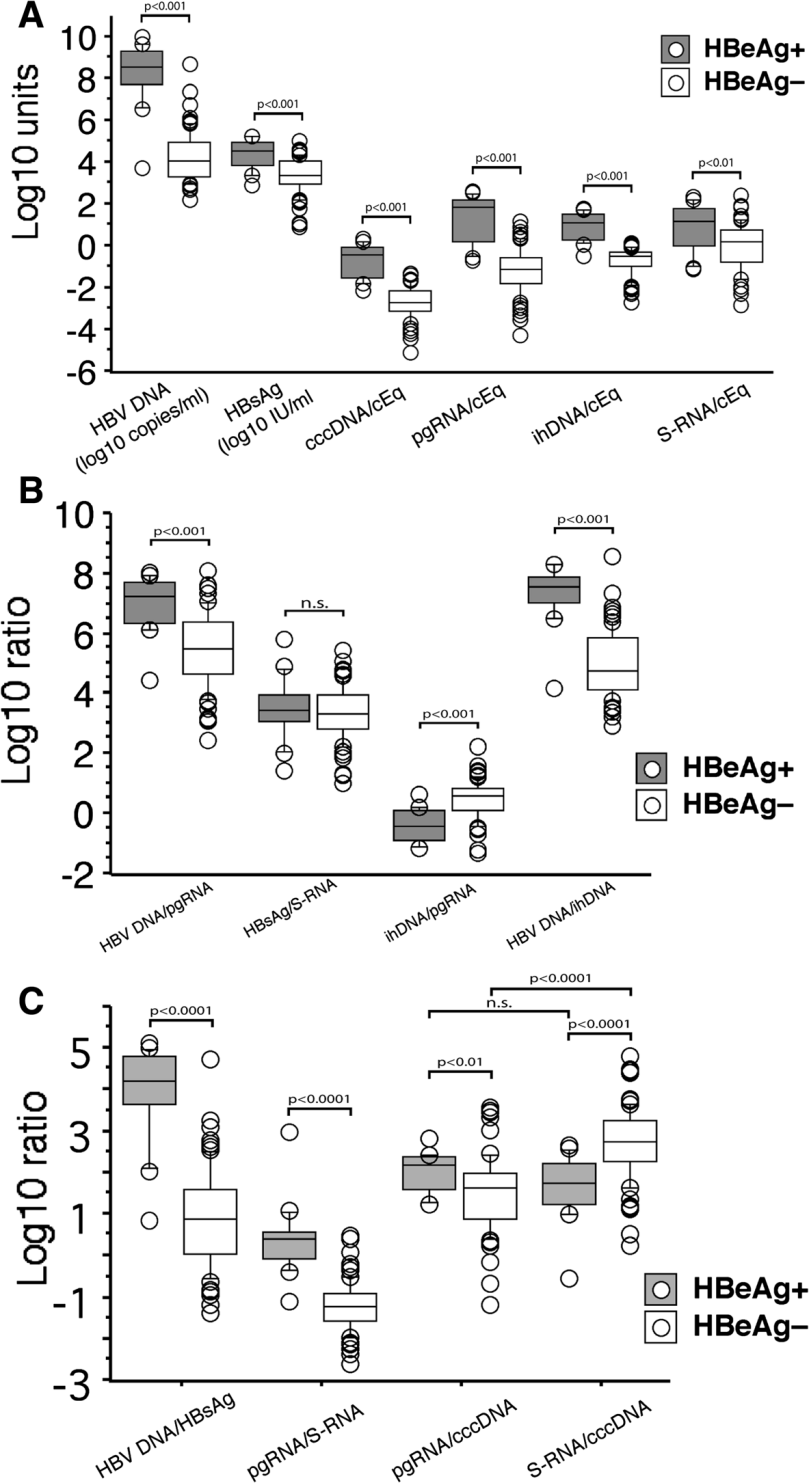
Box plots of serum and intrahepatic levels of hepatitis B virus markers in HBeAg-positive and HBeAg-negative patients (**a**) and their ratios (**b** and **c**). Each box shows median and 25^th^ and 75^th^ percentile, the bars 10^th^ and 90^th^ percentile, and open circles results outside the latter range. cEq, cell equivalent

Comparisons of viral markers at group level (HBeAg-positive vs. negative) revealed differences due to combined effects before and after loss of HBeAg, and are shown by box plots in Fig. 3a. Thus, the mean (median) cccDNA levels were 1.97 (2.27) log_10_ lower, pgRNA levels 2.52 (2.98) log_10_ lower, and serum HBV DNA levels 4.00 (4.50) log_10_ lower in HBeAg-negative as compared with HBeAg-positive patients. These differences influence the ratios between these parameters as shown in Fig. 3b. Thus, the HBV DNA/pgRNA and HBV DNA/ihDNA ratios were much lower, whereas the HBsAg/S-RNA ratio was the same, in HBeAg-negative as compared with HBeAg-positive patients.

### Difference in reduction of viral and subviral particles

This was studied by comparing HBV DNA and HBsAg levels in serum (i.e. the ratio between them), and by comparing the corresponding transcripts (pgRNA and S-RNA). As shown in Fig. 3a, the levels of HBsAg and S-RNA were only moderately lower in HBeAg-negative patients (mean 1.0, median 0.87 log_10_ for HBsAg; mean 1.24, median 1.72 log_10_ for S-RNA). The diverging degree of reduction of viral and subviral particles is further illustrated by Fig. 3c, which shows how ratios between levels of viral and subviral markers change after loss of HBeAg. Thus, there was a marked reduction of the HBV DNA/HBsAg ratio, and this was to some extent observed also for the corresponding transcripts, pgRNA/S-RNA. However, whereas the ratio pgRNA/cccDNA was lower after loss of HBeAg, the ratio S-RNA/cccDNA was instead increased. Thus, the pgRNA/cccDNA ratio was significantly lower than S-RNA/cccDNA in HBeAg-negative patients (*P* < 0.0001), but similar in HBeAg-positive patients.

## Discussion

This study of intrahepatic HBV RNA and HBV DNA and their correlation with each other and with levels of HBV DNA and HBsAg in serum may help to understand how HBV replication is suppressed during the course of infection and how low serum levels of HBV DNA and HBsAg are achieved. Part of our results agree well with published studies [11–13], but the observations of an increased S-RNA/pgRNA ratio after loss of HBeAg, and of the much greater decline of serum HBV DNA than of intrahepatic HBV DNA have to our knowledge not been described before.

Within HBeAg-positive patients, lower serum levels of HBV DNA essentially reflected lower levels of the cccDNA template in combination with reduced amounts of pgRNA per cccDNA. Our results, with a 2 log_10_ lower cccDNA level in HBeAg-negative patients are identical with the observations by Werle-Lapostelle et al. [11], whereas other studies have reported smaller reduction (≈1 log_10_) after loss of HBeAg [12, 13]. The reduced levels of cccDNA after loss of HBeAg is probably mainly a result of eradication of infected hepatocytes by cytotoxic T cells [14] in combination with lower cccDNA content in each infected cell.

Concern has been raised regarding the specificity of cccDNA PCR assay, i.e. whether this assay amplifies only cccDNA. When this method was introduced by Köck and Schlicht they showed that the degree of cross-reactivity, i.e. amplification by cccDNA primers of other forms of HBV DNA, was low, corresponding to a 3 log_10_ lower sensitivity [15]. We found the same degree of cross-reactivity when we repeated their experiments using real-time PCR (results not shown). When analysing liver biopsies, we found that cccDNA levels were on average 1.90 log_10_ lower than ihDNA, and not 3 log_10_ lower as one would expect if the cccDNA findings were due to cross-reactivity. This indicates that a low specificity of the assay should have limited impact on our cccDNA results.

In addition to lower cccDNA levels, we found in HBeAg-negative patients a lower pgRNA/cccDNA ratio. This mechanism of reduced virus production has been proposed by others, and might be mediated by different mechanisms such as accelerated intracellular clearance of pgRNA-containing capsids induced by IFN-alfa/-beta [16], or by IFN-alfa mediated suppression of HBV replication by epigenetic control of cccDNA function and transcription [17]. Among our patients, the reduction of the pgRNA/cccDNA ratio was quite moderate (0.57 log_10_), whereas Volz et al. and Laras et al. found larger reductions (0.88 and 1.46 log_10_) [12, 13].

Within HBeAg-positive stage, pgRNA and serum HBV DNA correlated strongly with a slope of ≈ 1, meaning that 1 log_10_ decline of pgRNA results in 1 log_10_ decline of HBV DNA in serum. In HBeAg-negative patients, the relations between cccDNA, replicative intermediates (pgRNA and ihDNA) and levels of HBV DNA in serum were more complex.

Firstly, levels of pgRNA showed poorer correlation with cccDNA, suggesting that suppression acting on this step varies considerably between patients.

Secondly, in patients with lower cccDNA there were relatively higher levels of ihDNA, as indicated by both the slope of the ihDNA/pgRNA regression line and the higher ihDNA/pgRNA ratio in HBeAg-negative patients. The explanation to this relative increase of ihDNA among HBeAg-negative patients is uncertain. It might be due to production of non-productive replicative intermediates, or retention of viral particles that are not secreted into the blood. The latter possibility would agree with a study describing that viral particles can be blocked by anti-HBs antibodies internalized in liver cells [18]. Another study did however not report differences in virion release between HBeAg-positive and negative patients [19], and our finding and possible explanations need to be further explored. One also has to consider the possibility that integrated HBV DNA contributes to ihDNA, because such integrations would be detected by our real-time PCR assays (if they include the target region). In early stage their contribution would probably be minimal, but when the production of relaxed circular DNA in virions declines in HBeAg-negative patients the amount of integrated HBV DNA might be high enough to influence the measured ihDNA level.

Thirdly, in several HBeAg-negative patients the HBV DNA level in serum was reduced much more than what would be expected by the reduction of ihDNA, suggesting that factors acting downstream of reverse transcription may have great impact on HBV DNA in serum. Such factors might, as mentioned above, be reduced release of virions from infected cells, or shorter half-life of viral particles in serum. The possibility that enhanced clearance of free virions from the blood contributes to the pronounced reduction of HBV DNA levels in serum after loss of HBeAg has not been much studied or discussed. Mathematical calculations based on HBV kinetics under antiviral therapy [20] suggested that the half-life of free virions is ~25 h in HBeAg-positive and ~13 h in HBeAg-negative patients [21]. Another study reported similar half-lives in HBeAg-positive patients (17 h), but extremely short half-life in HBeAg-negative patients (0.6 s) [19], data that subsequently were questioned [22]. Despite the discrepancies it seems likely that stronger immunologic responses may lead to faster clearance of virions, but how much this influences serum levels of HBV DNA remains to be elucidated.

Whereas HBV DNA levels were more than 4.0 log_10_ lower in HBeAg-negative patients, levels of HBsAg were on average only 1.1 log_10_ lower. Accordingly, the HBV DNA/HBsAg ratio was 3.3 log_10_ lower in HBeAg-negative patients, as observed also in previous studies [13, 23, 24]. To some extent this was explained by a reduced ratio (by 2.0 log_10_) between the corresponding transcripts (pgRNA/S-RNA) in HBeAg-negative patients. This reduction of pgRNA/S-RNA is greater than the 1.2 log_10_ difference reported by Volz et al. [13], and was explained by both reduced pgRNA/cccDNA (as mentioned above) and an increased S-RNA/cccDNA ratio by 1.0 log_10_. This finding suggests that the smaller reduction of HBsAg after loss of HBeAg than expected from the cccDNA decline might be due to an enhanced transcription of S-RNA from cccDNA, or possibly transcription of S-RNA from integrated sequences of HBV DNA that contain the complete S region, including the promoter. Such transcription occurs in the Alexander hepatoma cell line [25], but whether this type of integrations occur during natural infection remains to be demonstrated.

The ratio between HBsAg/S-RNA was unchanged after loss of HBeAg, indicating that post-transcriptional processing of S-RNA and turnover of subviral particles remain unchanged after loss of HBeAg.

## Conclusions

**Fig. 4:**
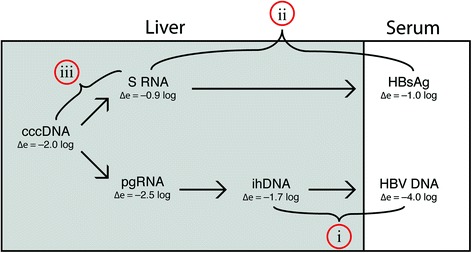
Flowchart showing differences in intrahepatic and serum viral load between HBeAg-positive and HBeAg-negative patients. Δe indicates mean difference between levels in HBeAg-positive and HBeAg-negative patients. Three novel findings are pointed out: (i) a greater reduction of HBV DNA in serum as compared with in the liver; (ii) an unchanged HBsAg/S RNA ratio; (iii) an increased S-RNA/cccDNA ratio, in HBeAg-negative as compared with HBeAg-positive patients

Figure 4 summarises the main findings in this study, pointing out three novel observations for HBeAg-negative patients:

i. A much lower HBV DNA/ihDNA ratio shows that mechanisms downstream of reverse transcription or downstream of secretion of virions strongly influence HBV DNA levels in serum.
ii. An unchanged HBsAg/S-RNA ratio suggests that secretion and degradation of SVP do not change after loss of HBeAg.
iii. An increased S-RNA/cccDNA ratio suggests that S-RNA and HBsAg might be produced from integrated S genes.

## Methods

### Patients

From a cross-sectional study of 160 patients with chronic HBV infection focusing on histology and HBV DNA levels [26] a total of 84 patients were included in this study, 16 positive and 68 negative for HBeAg. The present study is an extension of a previous study by our group [3]. From these patients liver biopsies were available for analysis by molecular techniques. None of the patients were co-infected with hepatitis C or D viruses, or HIV. The patients represented different genotypes and phases of HBV infection. Serum samples that were used for quantification of HBV DNA and HBsAg were taken at the time of biopsy. All patients gave informed consent and The Regional Ethical Review Board in Gothenburg approved the study.

### Laboratory assessments

Serum HBV DNA was analysed by Cobas Amplicor HBV Monitor (Roche Diagnostic Systems, Branchburg, NJ) and HBsAg in serum was quantified using the Architect assay (Abbott, Abbott Park, IL).

Portions (approximately 5 mg) of liver biopsies were investigated. The liver biopsies were stored in –70 °C until analysed. After homogenization of the liver tissue in a MagNA Lyser instrument (Roche Diagnostics), extraction of nucleic acids was performed in the MagNA Pure (Roche) robot according to the manufacturer’s protocol, using the DNA II Tissue kit.

Real-time PCR was performed using primers specific for cccDNA as previously described [3]. These primers amplify a segment spanning the gaps in the plus and minus strands of the relaxed circular form of the genome that is present in viral particles. Levels of cccDNA were normalised to the concentration of human betaglobin DNA. Total intrahepatic HBV DNA was analysed by the same primers as for pgRNA but without the reverse transcription step. Part of the extracted NAs was treated with DNase (Ambion Inc.), and S-RNA and pgRNA transcripts were quantified by real-time PCR after a reverse transcription step (details of the primers are shown in Table 2). Due to the overlap of S and pgRNA transcripts, the S primers and probe also detects pgRNA. Normalization of RNA levels was made using 18S RNA as reference.

**Table 2:** Oligonucleotide sequences of primers and probes

Target	Oligo	Sequence^a^	Nucleotide position^b^
S-RNA	Forward primer	TCCTCCAAYTTGTCCTGGTYATC	350–372
	Reverse primer	AGATGAGGCATAGCAGCAGGAT	432–410
	Probe (AS)	ATGATAAAACGCCGCAGACACATCCARC	400–373
pgRNA	Forward primer	GGTCCCCTAGAAGAAGAACTCCCT	2367–2390
	Reverse primer	CATTGAGATTCCCGAGATTGAGAT	2454–2431
	Probe	TCTCAATCGCCGCGTCGCAGA	2408–2428
cccDNA	Forward primer	CCGTGTGCACTTCGCTTCA	1575–1593
	Reverse primer	GCACAGCTTGGAGGCTTGA	1882–1864
	Probe	CATGGAGACCACCGTGAACGCCC	1607–1629

^a^Y, T or C; R, A or G; AS, antisense

^b^Position in genotype A genome

### Statistical analysis

Differences in levels of HBV RNA or DNA between groups were analysed by unpaired *t*-test. Correlations were made by linear regression and Pearson’s correlation analysis. *P* values below 0.05 were considered significant. The Statview software (SAS Institute) was used for statistical analyses.

## Abbreviations

cccDNA: covalently closed circular DNA
HBeAg: hepatitis B e antigen
HBsAg: hepatitis B surface antigen
HBV: hepatitis B virus
ihDNA: intrahepatic DNA
pgRNA: pregenomic RNA
